# Development of a triplex FMCA assay for genotyping three genes, *ADH1B*, *ADH1C*, and *ALDH2*, involved in alcohol metabolism

**DOI:** 10.1038/s41598-026-46895-y

**Published:** 2026-03-31

**Authors:** Mikiko Soejima, Yoshiro Koda

**Affiliations:** Department of Forensic Medicine, Kurume University School of Medicine, Kurume, 830- 0011 Japan

**Keywords:** Alcohol metabolism, Rs671, Rs698, Rs1229984, Fluorescence melting curve analysis, Multiplex genotyping, Biomarkers, Cancer, Computational biology and bioinformatics, Genetics, Molecular biology, Oncology

## Abstract

Three functional single nucleotide variants (SNVs)‒*ALDH2* rs671 (p.E504K), *ADH1C* rs698 (p.I350V), and *ADH1B* rs1229984 (p.R48H)‒are key genetic determinants of human alcohol metabolism. These variants significantly affect drinking behavior and are associated with liver disease and increased risks of several malignancies, including esophageal and gastric cancers. We developed a triplex fluorescent probe-based melting curve analysis (FMCA) assay for the simultaneous detection of these three SNVs. The assay was validated by comparing FMCA results with Sanger sequencing using genomic DNA from 94 Japanese individuals. The automated detection algorithm reliably identified genotypes of rs671 and rs698. Although the melting peaks of rs1229984 exhibited lower resolution and necessitated manual visual inspection for definitive genotype discrimination, all genotypes were nevertheless correctly identified. The assay demonstrated 100% accuracy. In conclusion, this triplex FMCA assay provides a rapid, cost-effective, and streamlined method for the simultaneous genotyping of *ADH1B*, *ADH1C*, and *ALDH2*. Given its high accuracy and ease of implementation, this method serves as a practical alternative to conventional sequencing, positioning it as a valuable tool for both large-scale epidemiological research and routine clinical assessment of alcohol-related health risks.

**Supplementary Information:**

The online version contains supplementary material available at 10.1038/s41598-026-46895-y.

## Introduction

Ethanol is metabolized through a sequential oxidative pathway in which alcohol dehydrogenase (ADH) converts ethanol to acetaldehyde, followed by aldehyde dehydrogenase (ALDH)–mediated oxidation of acetaldehyde to acetic acid^1,2^. Interindividual variation in the catalytic efficiency of these enzymes affects alcohol consumption patterns, susceptibility to alcohol dependence, the development of alcohol-associated liver disease, and the severity of physiological responses after alcohol intake^3,4^. Because both ethanol and acetaldehyde are classified as human carcinogens, elucidating the genetic determinants of alcohol metabolism is essential for understanding the etiology of several cancers including esophageal and gastric cancers. Importantly, the risk of these malignancies is not determined by a single locus but is substantially influenced by the synergistic interactions of multiple functional variants^5–7^. Therefore, a comprehensive assessment of these risks‒rather than analyzing individual single nucleotide variants (SNVs) in isolation‒is crucial for identifying high-risk individuals and implementing personalized cancer prevention strategies.

Among the loci involved in ethanol metabolism, *ADH1B* (rs1229984, p.R48H), *ADH1C* (rs698, p.I350V), and *ALDH2* (rs671, p.E504K) are well-established nonsynonymous SNVs with clear functional consequences^5–7^. The A alleles of rs1229984 (ADH1B*2) and rs698 (ADH1C*1) encode ADH isoforms with markedly high catalytic turnover, whereas the corresponding G alleles (ADH1B*1, ADH1C*2) produce low-activity isoforms^8,9^. Individuals carrying low-activity ADH variants exhibit slower ethanol-to-acetaldehyde conversion, resulting in prolonged systemic ethanol exposure. Recent large-scale genomic studies and global burden assessments have reinforced this factor as a critical driver of increased carcinogenic risk, not only for the aero-digestive tract but also for various systemic malignancies and other alcohol-related disorders^10–13^. Reduced acetaldehyde accumulation also diminishes aversive physiological responses, thereby facilitating higher alcohol intake and increasing vulnerability to alcohol dependence^14^. The rs1229984 A allele is highly enriched in East Asian populations‒including Japanese, Chinese, and Koreans‒where its frequency approaches 70%, but it remains uncommon (< 10%) in Europeans, Africans, and South Asians. In contrast, the rs698 A allele is prevalent in East Asians and Africans (80–90%) and moderately frequent in Europeans (~ 60%)^5^.

*ALDH2* rs671 also exhibits pronounced population stratification. The G allele (ALDH2*1) encodes an enzyme with robust acetaldehyde-detoxifying capacity, whereas the A allele (ALDH2*2) produces an enzyme with severely diminished (G/A heterozygote) or absent (A/A homozygotes) activity^15^. Individuals with low or null ALDH2 function accumulate high levels of acetaldehyde after alcohol intake, leading to characteristic alcohol-induced reactions such as facial flushing, headache, and nausea, and markedly increasing the risk of upper gastrointestinal carcinogenesis^16^. In East Asian populations, the rs671 A allele frequency ranges from 20% to 30% (Source: dbSNP, https://www.ncbi.nlm.nih.gov/snp/rs671). Specifically, among Japanese individuals, approximately 37% carry the low-activity G/A heterozygous genotype and ~ 7% carry the null-activity A/A homozygous genotype^17^. In contrast, more than 90% of non-East Asian populations‒including Europeans, Africans, and Indians‒are homozygous for the high-activity G allele^5^.

Given these striking interpopulation differences and their substantial implications for alcohol-related disease susceptibility, a rapid and reliable genotyping platform for these three SNVs would be highly useful for assessing drinking behavior, alcohol-related liver disease, and carcinogenic risk. However, current PCR-based methods such as Sanger sequencing suffer from limited throughput or high cost when analyzing multiple loci^18^.

To overcome the limitations of conventional assays, TaqMan genotyping assays, which employ dual-labeled hydrolysis probes, are widely used for SNV discrimination^19^. During amplification, the 5’−3’ exonuclease activity of Taq polymerase cleaves the probe, generating a fluorescence signal. Alternatively, fluorescence melting curve analysis (FMCA) has emerged as a high-resolution method for SNV identification^20^. Because FMCA does not rely on probe degradation, it is compatible with polymerases regardless of their 5’−3’ exonuclease activity. A key advantage of FMCA over TaqMan assays is that only a single probe is required per SNV, enabling efficient multiplex genotyping within a single reaction^20–23^.

Recently, a genotyping method using FMCA for *ALDH2* rs671 was reported. However, this method is limited to the detection of a single SNV^24^. Therefore, in this study, we developed a triplex FMCA platform capable of simultaneously genotyping rs671, rs698, and rs1229984 with high analytical performance. This platform provides a practical tool for relatively large-scale screening in various populations, particularly East Asians, among whom the synergistic effects of these alcohol-metabolizing gene variants significantly impact public health.

## Results

### Genotypes of rs671, rs698, and rs1229984 determined by Sanger sequencing

To compare the results of FMCA, we first determined the genotypes of *ALDH2* (rs671), *ADH1C* (rs698), and *ADH1B* (rs1229984) in 94 Japanese subjects by Sanger sequencing of the PCR products. Raw sequence data were deposited in the DDBJ repository (BioProject PRJDB40219; Run accessions DRR902565-DRR902658). The quality and integrity of the newly extracted DNA were verified by agarose gel electrophoresis, which showed distinct high-molecular-weight bands. While slight degradation was observed in several samples, the overall DNA quality was deemed sufficient for downstream PCR and FMCA applications (Supplementary Figure S1). Furthermore, Sanger sequencing yielded clear and robust electropherograms for all 94 samples, consistently confirming the suitability of the template DNA, as well as the sufficient amplification efficiency and specificity of the designed primers. As described in the Materials and methods, in the method designed here, the alleles of rs698 and rs1229984 are represented as C and T instead of G and A (Table 1).

**Table 1:** Primer and probe sequences used in this study.

SNP	Probe/Primer Name	Sequence (5’→3’)
rs671	ALDH2-F primer	GGCTACAAGATGTCGGGGAG
	ALDH2-R primer	CCACCAGCAGACCCTCAAG
	ALDH2-probe	[Cy5]-GGCATACACTGAAGT*G*AAAAC-[BHQ-2]
rs698	ADH1C-F primer	CGAAGCAGGTCAAATCCTTCA
	ADH1C-R primer	TCCCCAAACTTGTGGCTGAC
	ADH1C-probe	[HEX]-AGGTAAAA*C*ATTTGTTATTAATGCA-[BHQ-1]
rs1229984	ADH1B-F primer	TCCAACACTCTCCACGATGC
	ADH1B-R primer	TCTGAATCTGAACAGCTTCTCTT
	ADH1B-probe	[FAM]-GGTCATCTGTG*C*GACAGATT-[BHQ-1]

Positions of rs671, rs698, and rs1229984 are underlined. For rs698 and rs1229984, C/T alleles are defined based on the reverse complement of the mRNA sequence to match the probe orientation used in this assay. *BHQ* Black Hole Quencher.

The genotype and allele frequencies are summarized in Table 2. In our study population, the frequencies of the inactive ALDH2*2 (A) allele and the rapid-metabolizing ADH1B*2 (T) allele were 25.5% and 73.4%, respectively. Specifically, 5.3% of individuals (*n* = 5) possessed the ALDH2*2 homozygote (A/A), while 73.4% (*n* = 69) carried the ADH1B*2 homozygote (T/T). To assess the genetic representativeness and data quality of our cohort, the genotype distributions of the three SNVs were tested for Hardy–Weinberg equilibrium (HWE) using the chi-square test. All variants‒rs671, rs698, and rs1229984‒showed no significant deviation from HWE (*p* = 0.5407, 0.8691, and 0.7261, respectively). Furthermore, the minor allele frequencies observed in our study population were highly comparable to those reported for East Asian populations in the dbSNP database (Source: https://www.ncbi.nlm.nih.gov/snp/): rs671 (0.255 vs. 0.225), rs698 (0.096 vs. 0.070), and rs1229984 (0.245 vs. 0.296). These results confirm the genetic representativeness and reliability of our study cohort.

**Table 2:** Genotype and allele frequencies of *ALDH2* (rs671), *ADH1C* (rs698), and *ADH1B* (rs1229984) in 94 Japanese subjects.

rs671		rs698		rs1229984	
G/G	51/94 (54.3%)	C/C	1/94 (1.1%)	C/C	5/94 (5.3%)
G/A	38/94 (40.4%)	C/T	16/94 (17.0%)	C/T	36/94 (38.3%)
A/A	5/94 (5.3%)	T/T	77/94 (81.9%)	T/T	53/94 (56.4%)
G allele	140/188 (74.5%)	C allele	18/188 (9.6%)	C allele	46/188 (24.5%)
A allele	48/188 (25.5%)	T allele	170/188 (90.4%)	T allele	142/188 (75.5%)

The probe sequences for rs698 and rs1229984 are complementary to the coding strand (mRNA sequence). Therefore, C and T alleles indicate low and high alcohol metabolism activities, respectively.

### Optimization of triplex FMCA conditions

**Fig. 1:**
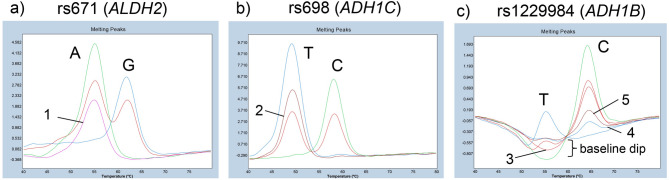
Representative melting peak profiles for the genotyping of rs671 (**a**), rs698 (**b**), and rs1229984 (**c**). The automated software categorized genotypes into three primary clusters: green, blue, and red (refer to individual panels for genotype assignments). Arrows indicate specific instances of automated misclassification that required manual resolution: (1) an rs671 A/A subject misidentified as “Group 4” (pink); (2) an rs698 T/T subject misidentified as “Unknown” (brown). For rs1229984 (C), subject (3) (genotype C/C) was misidentified as C/T (red), while subjects (4) and (5) (genotype C/T) were misidentified as T/T (blue) and “Unknown” (brown), respectively.

We then optimized PCR and FMCA conditions to enable simultaneous detection of these three SNVs in a single triplex FMCA assay. Based on preliminary testing of multiple DNA polymerases, Probe qPCR Mix MultiPlus was selected for the final protocol due to its superior robustness and reproducibility in generating distinct, sharp melting peaks. Under default analysis settings, the genotypes of rs671 and rs698 were clearly distinguishable. For rs671, the *T*_*m*_ values were approximately 62 °C for G/G, 55 °C and 62 °C for A/G, and 55 °C for A/A (Fig. 1a). For rs698, the *T*_*m*_ values were approximately 58 °C for C/C, 49 °C and 58 °C for C/T, and 49 °C for T/T (Fig. 1b).

### Challenges in automated genotyping and accuracy of rs1229984

Compared to rs671 and rs698, rs1229984 exhibited more complex melting curve characteristics. FMCA revealed two peaks corresponding to the C allele (*T*_*m*_ approximately 65 °C) and the T allele (*T*_*m*_ approximately 56 °C). However, the peak intensities and baseline behaviors differed significantly between genotypes (Fig. 1c).

In C/C homozygotes, while the higher-temperature peak was prominent, a subtle baseline dip was frequently observed at the expected position of the T-allele peak. In C/T heterozygotes, the lower-temperature peak (T-allele) exhibited an upwardly convex shape but with markedly low intensity compared to the C-allele peak. Therefore, as shown in Fig. 1c, C/T heterozygotes with low C-allele peak intensity were often categorized as “unknown” or incorrectly assigned as T/T homozygotes even under modified analysis settings (score threshold 0.30, resolution threshold 0.15, normal sensitivity). In addition, some C/C homozygotes with low peak intensity were also misclassified as C/T heterozygotes (Fig. 1c), and negative controls were misidentified as T/T homozygotes due to minor baseline fluctuations. Notably, while negative controls exhibited only irregular baseline noise without any distinct thermal transitions, the T-allele in sample DNA consistently formed a discernible upward-pointing peak, even at low intensities.

Crucially, despite these automated miscalls, each genotype possessed a consistent “morphological signature” in its melting profile. The T-allele peak in heterozygotes, although low in amplitude, maintained a reproducible derivative shape that was visually distinguishable from the baseline noise or dips seen in C/C samples. By performing manual visual inspection focused on these curve characteristics, we were able to resolve all automated ambiguities. By employing this hybrid approach—integrating automated clustering with expert visual confirmation—we ensured 100% concordance with Sanger sequencing results for rs1229984, demonstrating that our validated workflow remains robust even when automated algorithms reach their limits.

### Quantitative performance and error rates

Low-intensity peaks also caused rare automated misidentifications for rs671 and rs698 (Fig. 1a and b), though these occurred much less frequently than for rs1229984. Table 3 summarizes the error rates across four independent triplex FMCA experiments.

**Table 3:** Genotyping error rates across four independent FMCA runs.

SNVs	Genotype	No.	1st	2nd	3rd	4th	Error rates
rs671	G/G	51	0	0	0	0	0/204 (0.0%)
	G/A	38	0	0	0	0	0/152 (0.0%)
	A/A	5	0	0	0	1^a^	1/20 (5.0%)
	total	94	0	0	0	1	1/376 (0.3%)
rs698	C/C	1	0	0	0	0	0/4 (0.0%)
	C/T	16	0	0	0	0	0/64 (0.0%)
	T/T	77	0	0	0	1^b^	1/308 (0.3%)
	total	94	0	0	0	1	1/376 (0.3%)
rs1229984	C/C	5	1	1	1	2	5/20 (25%)
	C/T	36	1	2	5	3	11/144 (7.6%)
	T/T	53	0	0	0	0	0/212 (0.0%)
	total	94	2	3	6	5	16/376 (4.3%)

As detailed in the previous section, rs1229984 C/C homozygotes exhibited the highest automated misclassification rate (25.0%; 5/20 tests), primarily due to the software recognizing baseline fluctuations as heterozygous peaks. Additionally, 7.6% of C/T heterozygotes (11/144 tests) were misclassified as T/T or labeled as “unknown” due to the inherently low intensity of the T-allele peak. In contrast, rs671 and rs698 demonstrated exceptional reliability, with automated error rates of only 0.3% (1/376 tests) each.

**Fig. 2:**
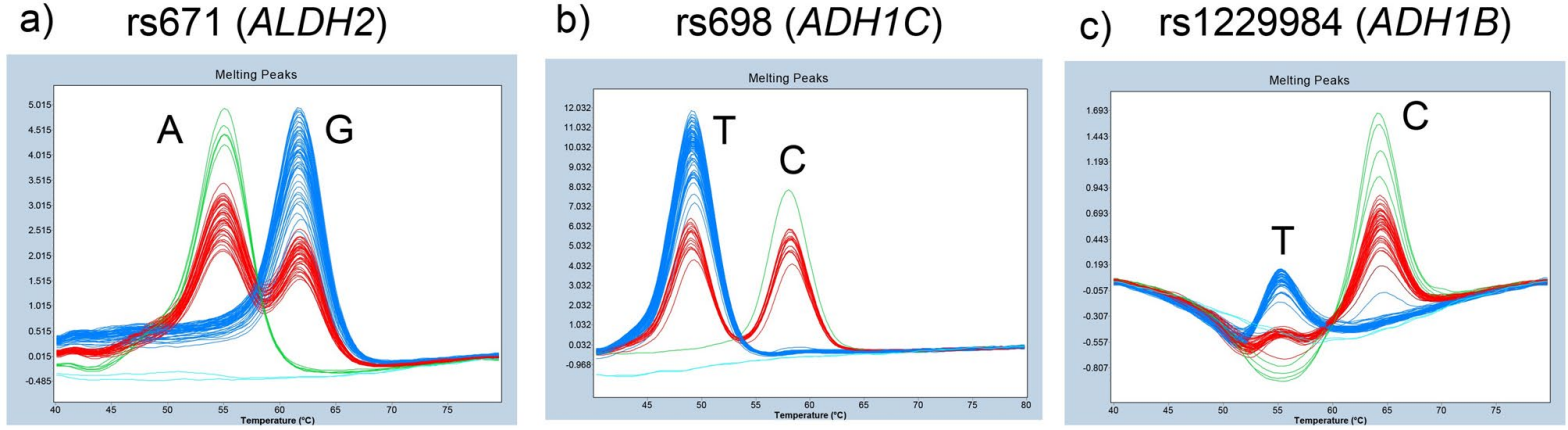
Comprehensive triplex FMCA profiles of 94 Japanese subjects across four independent experiments. **a** rs671 (*ALDH2*): genotypes A/A (green), A/G (red), and G/G (blue) are clearly clustered. **b** rs698 (*ADH1C*): genotypes T/T (blue), C/T (red), and C/C (green) are well-differentiated. **c** rs1229984 (*ADH1B*): genotypes are shown as T/T (blue), C/T (red), and C/C (green). Light blue lines in all panels represent negative controls. Notably, the automated software misidentified the negative control in **(c)** as T/T due to baseline fluctuations. One subject with genotype C/C was misclassified as “Unknown” (brown), and one subject with C/T was misidentified as T/T (blue); both cases were corrected through manual visual inspection.

Notably, the vast majority of these rare errors for rs671 and rs698 resulted in “unknown” or “Group 4” (unclassified) labels rather than the assignment of an incorrect valid genotype. Because these samples are automatically segregated into distinct error categories by the software, they are easily flagged for subsequent manual review. This characteristic minimizes the risk of overlooking genotype miscalls and ensures the high diagnostic fidelity of the assay. Representative melting curve profiles demonstrating the consistency of these profiles across four independent experiments are shown in Fig. 2a–c.

## Discussion

In this study, we developed a triplex FMCA method capable of simultaneously detecting three alcohol metabolism-related SNVs‒*ALDH2* (rs671), *ADH1C* (rs698), and *ADH1B* (rs1229984)‒within a single reaction. Under default analysis settings, rs671 and rs698 were automatically detected with high accuracy, with only one misclassification each observed among 376 reactions. These rare errors were attributable to low-intensity peaks and were categorized by the automated system as “unknown” or assigned to an additional cluster distinct from the three valid genotypes, allowing straightforward correction through visual inspection.

In contrast, rs1229984 exhibited the substantially higher automated misclassification rate of approximately 4.3%. Unlike rs671 and rs698, miscalls for rs1229984 frequently involved assignment to an incorrect valid genotype rather than “unknown” or assigned to an additional cluster. This behavior appears to stem from the unique melting curve morphology of rs1229984. Specifically, C/C homozygotes displayed a baseline dip at the expected T-allele position, while C/T heterozygotes exhibited an extremely low intensity T-allele peak. These features likely interfered with the automated clustering algorithm, leading to misrecognition. Nevertheless, because the characteristic curve shapes remain visually distinguishable, accurate genotype calling is readily achievable through manual inspection.

An important observation in this study was that samples exhibiting low peak intensity varied across experiments. This indicates that low peaks are not attributable to specific sample conditions. While agarose gel electrophoresis revealed slight degradation in several samples (Supplementary Figure S1), these specific samples did not consistently show poor FMCA performance. Instead, the variations appear to arise from stochastic factors inherent to FMCA, such as minor fluctuations in PCR efficiency, subtle temperature variations, and slight differences in reaction composition. Therefore, for multiplex SNV detection using FMCA, incorporating retesting and visual confirmation when necessary is effective for ensuring reproducibility and reliability.

This study also highlighted a weakness in the current automated detection algorithm. FMCA-based automated calling relies on clustering based on peak position, height, and curve shape; however, fixed threshold settings may not adequately accommodate SNV-specific curve characteristics or low-intensity peaks. SNVs such as rs1229984, which inherently exhibit weaker signals, are particularly susceptible to misclassification. The observed baseline dip and low peak intensity for the rs1229984 T-allele may be attributed to the specific sequence context or thermodynamic instability of the probe-target duplex at that locus. While this poses a challenge for current automated clustering, the distinct ‘morphological signature’ of the melting curves ensures high fidelity in manual calling. Future improvements to the automated algorithm could incorporate pre-processing techniques, such as baseline subtraction or normalization to internal references, to stabilize peak heights and minimize the impact of the observed baseline fluctuations. Furthermore, the integration of machine learning-based signal processing—potentially utilizing a two-stage calling rule with quality control flags—could eliminate this manual step, further streamlining the workflow for ultra-high-throughput clinical applications.

Additionally, FMCA offers several advantages, including the ease of multiplexing, rapid turnaround time, and low cost. However, the results also demonstrate that detection performance depends on enzyme selection and probe design. In this study, Probe qPCR Mix MultiPlus produced the most robust and reproducible results among the tested polymerases. This is consistent with our previous experience in developing other triplex FMCA assays^22^, suggesting that this specific enzyme mix may be particularly well-suited for the thermodynamic demands of multiplex FMCA. While further validation across a broader range of SNV targets will be beneficial to confirm its versatility, future studies should also focus on defining standardized analysis parameters—including temperature ramp rates and baseline regions—to ensure cross-platform reproducibility. In this context, the choice of a high-performance polymerase appears to be a critical factor for successful multiplexing and maintaining consistent performance across different laboratory environments. Additionally, incorporating probes that achieve higher *T*_*m*_ values even with short sequences, such as Minor Groove Binder or Locked Nucleic Acid probes^25,26^, may further improve the detection of challenging SNVs like rs1229984. For instance, Minor Groove Binder-conjugated probes have been shown to enhance signal-to-noise ratios and stabilize duplexes in AT-rich regions^27^, while Locked Nucleic Acid modifications can significantly broaden the *T*_*m*_ difference (Δ*T*_*m*_) between alleles, facilitating clearer automated genotype clustering in FMCA assays^28^. Furthermore, optimizing the probe orientation might enhance the signal-to-noise ratio for challenging targets like rs1229984. In this study, probes for rs698 and rs1229984 were designed based on the reverse-complement strand. Future comparative studies evaluating probes complementary to the sense (mRNA) strand may reveal whether orientation-specific sequence contexts can improve the distinctness of the T-allele melting peak, thereby facilitating more robust automated genotype calling.

The ability to simultaneously analyze *ADH1B*, *ADH1C*, and *ALDH2* genotypes has significant implications for clinical and epidemiological research. These SNVs are strongly associated with drinking behavior, alcohol dependence, and risks of esophageal cancer, gastric cancer, and alcohol-related liver injury. Notably, previous studies have demonstrated that the synergistic interaction between the rapid-activity ADH1B*2 allele (T allele in this study) and the inactive ALDH2*2 allele (A allele in this study) dramatically elevates the risk of esophageal cancer‒by more than several hundred-fold in heavy drinkers compared to those without these variants^29^, and were recently confirmed by meta-analysis^11^. By integrating these critical loci into a single assay, our FMCA-based method ensures the reliable identification of such ultra-high-risk individuals without the need for multiple independent tests. This makes it an exceptionally efficient tool for stratifying disease risk in clinical settings. Triplex FMCA is therefore useful for determining genotype frequencies in large populations, stratifying disease risk, and conducting population genetic studies, including regional allele frequency gradients.

Despite the clinical utility of this triplex FMCA assay, several limitations should be acknowledged. First, although the assay demonstrated 100% concordance with Sanger sequencing in this study, the validation was conducted on a relatively small cohort of 94 Japanese individuals. While we observed specific allelic combinations associated with high acetaldehyde exposure (e.g., ADH1B*2 and ALDH2*2), this sample size may be insufficient to reliably infer the exact population frequencies of such rare co-occurrences in the broader Japanese population. Further validation with larger and more diverse East Asian populations is necessary to confirm the robustness of the assay across different genomic backgrounds. Second, the current necessity for manual inspection of rs1229984 signals may present a bottleneck in fully automated, ultra-high-throughput environments, as it introduces a minor degree of interpretive subjectivity. As previously mentioned, refining the probe chemistry or integrating more sophisticated signal-processing algorithms —such as machine learning-based protocols— would be essential to enhance objectivity and scalability. Finally, while these three SNVs are major contributors to alcohol metabolism, they do not account for the entire spectrum of alcohol-related disease risk. Environmental factors, such as total alcohol consumption and smoking, also play critical roles; therefore, this assay should be integrated into a broader, multifaceted risk assessment strategy.

While Sanger sequencing remains the gold standard for SNV analysis, its low throughput and higher operational costs limits its utility for large-scale studies. In our setting, the reagent cost for this triplex FMCA assay is estimated to be less than well below 0.65 USD (less than 100 JPY) per sample, and this cost could be further reduced by increasing the scale of probe synthesis. This represents a substantial saving compared to the multiple PCR, purification, and sequencing reactions required to identify three separate SNVs via the Sanger method. TaqMan assays offer high specificity but are constrained in multiplexing capacity. Next-generation sequencing provides comprehensive genomic information^30^, but is excessive and costly for rapid detection of a small number of SNVs. In contrast, FMCA utilizes standard real-time PCR instrumentation and a straightforward reaction setup^18^, making it an exceptionally practical and scalable platform for multiplex SNV detection across different laboratory settings. The rapid turnaround time of approximately 1.5 h from the start of PCR to the completion of melting curve analysis for a 96-well plate further enhances its suitability for high-throughput applications, making it one of the most practical and scalable options for multiplex SNV detection.

Furthermore, the low cost and rapid turnaround time of this assay make it an ideal tool for population-based cancer screening programs and routine health check-ups. Instead of relying solely on drinking frequency questionnaires, which are often subject to recall bias, clinicians can use this genetic data to provide evidence-based counseling. Identifying ‘high-risk’ genotypes before the onset of disease could facilitate early interventions, such as intensive lifestyle modifications or prioritized endoscopic surveillance, ultimately reducing the burden of alcohol-related malignancies in high-risk populations like East Asians.

## Conclusion

This study established a triplex FMCA method for the simultaneous genotyping of *ADH1B* rs1229984, *ADH1C* rs698, and *ALDH2* rs671 in a single reaction. While rs1229984 requires visual inspection due to unique melting peak characteristics, the assay reliably identifies all genotypes with high precision. This rapid and cost-effective platform serves as a practical tool for large-scale screening and personalized cancer risk stratification, potentially contributing to the prevention of alcohol-related malignancies.

## Materials and methods

### Ethical statements and DNA samples

All methods were carried out in accordance with relevant guidelines and regulations, including the Declaration of Helsinki. This study utilized existing anonymized genomic DNA samples from 94 randomly selected healthy Japanese individuals, as previously described^31^. Although the peripheral blood samples were originally collected in 1995, they had been continuously stored at −80 °C. For the present study, genomic DNA was re-extracted in 2021 using the QIAamp DNA Blood Mini Kit (QIAGEN, Hilden, Germany) to ensure high DNA integrity. The quality and concentration of the extracted DNA were assessed using BioSpec-nano (Shimadzu, Kyoto, Japan) and further verified by agarose gel electrophoresis (Supplementary Figure S1). At the time of collection in 1995, verbal informed consent for comprehensive genetic polymorphism analysis was obtained, which was the standard accepted practice in Japan at that time. The consent procedure and its verbal nature were explicitly approved by the Ethical Committee of Kurume University. To ensure participant privacy, all samples were strictly de-identified in an unlinkable manner before use. The protocol for the current study, which involves the use of these existing anonymized samples as well as commercially available genomic DNA, was initially approved by the Ethical Committee of Kurume University in 2002. Since then, the protocol has been renewed every five years, with the most recent approval granted on October 31, 2022 (Approval No. 22158).

### Probes and primers

The three SNVs‒rs671, rs698, and rs1229984‒were genotyped using specific PCR primer sets and fluorophore-labeled probes (Table 1). PCR primers were designed using Primer3Plus (source: https://www.bioinformatics.nl/cgi-bin/primer3plus/primer3plus.cgi)^32^. In addition, the thermodynamic properties and potential secondary structures of the designed oligonucleotides‒including melting temperature (*T*_*m*_) self-dimerization, and hairpin formation‒were verified using OligoCalc (Source: http://oligocalc.eu/)^33^. For the optimization of the rs1229984 assay, multiple probe designs were evaluated, including variations in probe length and testing both sense and antisense orientations, to achieve the most stable and distinct melting peaks within the triplex format.

All oligonucleotides, including FAM-, HEX-, and Cy5-labeled probes with appropriate quenchers (black hole quencher 1/black hole quencher 2), were custom-synthesized by Eurofins Genomics (Tokyo, Japan).

For rs698 and rs1229984, the target genomic regions corresponded to the reverse complement of the mRNA sequence. Consequently, in this assay, the C and T alleles represented the low-activity and high-activity alcohol metabolism phenotypes, respectively. It is important to note that for these two SNVs, the T allele in this assay corresponds to the A allele in the standard genomic forward strand nomenclature. This orientation was maintained throughout the study to ensure consistent data interpretation.

### Genotyping of rs671, rs698, and rs1229984 by Sanger sequencing

To determine the genotypes of rs671, rs698, and rs1229984 in this study, PCR products from all 94 individuals were subjected to direct Sanger sequencing. The target regions were amplified using specific primers (Table 1) in a 10 µL reaction mixture containing 1–10 ng of genomic DNA, 5 µL of Premix Ex Taq (Probe qPCR) (Takara Bio, Shiga, Japan), and 250 nM of each primer. The thermal profile comprised an initial denaturation at 95 °C for 30 s, followed by 45 cycles of denaturation at 95 °C for 5 s and annealing/extension at 60 °C for 15 s. The same primers used for PCR were employed for the sequencing reactions, which were outsourced to Eurofins Genomics. Sanger sequencing served as the reference method to validate the genotyping results obtained via FMCA.

### Real-time PCR monitoring and FMCA

Asymmetric PCR followed by fluorescence melting curve analysis (FMCA) was conducted for rs671, rs698, and rs1229984 using the LightCycler 480 II system (Roche Diagnostics, Tokyo, Japan). Each 10 µL reaction contained Probe qPCR Mix MultiPlus (Takara Bio) and 1–10 ng of genomic DNA. Primer and probe concentrations were optimized for each SNV. For rs671 and rs698, concentrations were 50/500/200 nM, while for rs1229984, they were 25/250/100 nM (forward/reverse/probe). In both cases, a 10-fold excess of reverse primers was maintained to promote asymmetric amplification^20,22^.

Thermal cycling conditions consisted of an initial denaturation at 95 °C for 20 s, followed by 45 cycles of 95 °C for 1 s and 60 °C for 20 s. Fluorescence signals were monitored using FAM, VIC/HEX/Yellow555, and Cy5/Cy5.5 filter sets during both the amplification phase and the subsequent melting curve analysis (40–80 °C at a ramp rate of 0.10 °C/sec). The real-time PCR instrument was calibrated according to the manufacturer’s instructions to ensure optical accuracy and minimize crosstalk between fluorescence channels.

The *T*_*m*_ values and genotypes were analyzed using LightCycler 480 Gene Scanning Software (v1.5). Default analysis parameters (score threshold 0.70, resolution threshold 0.10, normal sensitivity) were applied to rs698 and rs671. For rs1229984, which exhibited lower peak intensity and a distinct curve morphology, modified settings (score threshold 0.30, resolution threshold 0.15, normal sensitivity) were used to improve allele discrimination. Automated calls were generated based on clustering algorithms, followed by manual visual inspection to ensure definitive genotype discrimination, particularly for samples with low peak intensities. The reagent cost for this triplex FMCA assay was estimated to be less than 0.65 USD per sample, and the total turnaround time from the start of PCR to the completion of melting curve analysis was approximately 1.5 h.

## Supplementary Information

Below is the link to the electronic supplementary material.

Supplementary Material 1

## Data Availability

The datasets generated and analyzed during the current study are available in the DDBJ repository under BioProject accession number PRJDB40219 and Run accession numbers DRR902565-DRR902658.

## Abbreviations

ADH1B: Alcohol dehydrogenase 1B
ADH1C: Alcohol dehydrogenase 1C
ALDH2: Aldehyde dehydrogenase 2
FMCA: Fluorescence melting curve analysis
*T*_*m*_: Melting temperature

## Code availability

No custom code or mathematical algorithms were used to generate the results in this study. Data analysis was performed using the standard software provided by the manufacturer (LightCycler 480 Gene Scanning Software, version 1.5; Roche Diagnostics, Tokyo, Japan).
