# Supplementary material for: Development of a triplex FMCA assay for genotyping three genes, ADH1B, ADH1C, and ALDH2, involved in alcohol metabolism
Source: Sci Rep. 2026 Mar 31;16:15229. doi: 10.1038/s41598-026-46895-y (PMC13181127; doi:10.1038/s41598-026-46895-y)
Supplement: Supplementary file 1 — Supplementary Material 1 [file 41598_2026_46895_MOESM1_ESM.pdf]

Supplementary Figure S1

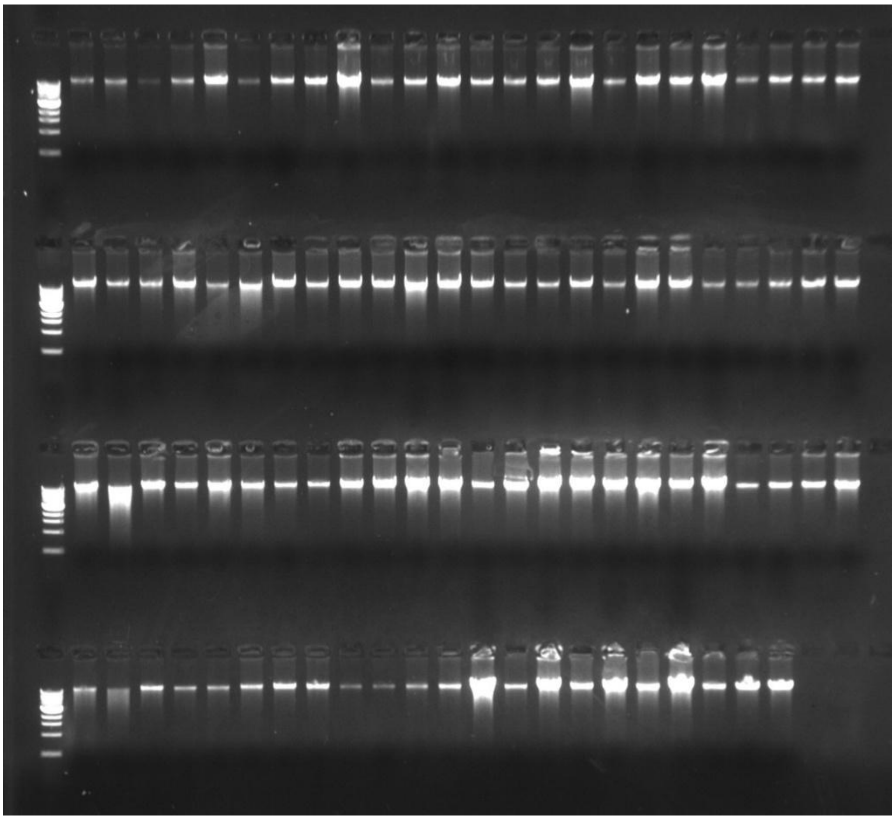

Supplementary Figure S1. Agarose gel electrophoresis of extracted genomic DNA. Genomic DNA from 94 Japanese individuals was visualized on a 1% agarose gel to assess quality and integrity. The molecular weight marker, NEB 1 kb DNA Ladder (size range: 0.5–10 kb), was loaded in the leftmost lane of each row. Samples are arranged as follows: 1–24 (top row), 25–48 (second row), 49–72 (third row), and 73–94 (bottom row). The presence of distinct bands positioned well above the 10 kb marker in most samples confirms the integrity of the high-molecular-weight genomic DNA. While slight degradation (smearing) was visible in several samples, the 100% concordance with Sanger sequencing results and the consistent FMCA performance across independent experiments (as discussed in the text) validated that this minor degradation did not adversely affect the genotyping accuracy.
